# Case Report: Imaging and pathological analysis of intra-abdominal aggressive fibromatosis with abscess

**DOI:** 10.3389/fonc.2025.1692823

**Published:** 2025-11-06

**Authors:** Rong Yang, Juan Zhao, Lin Zhou

**Affiliations:** 1 Department of Ultrasound, Suining Central Hospital, Suining, China; 2 Department of Pathology, Suining Central Hospital, Suining, China; 3 Department of Radiology, The 3rd Affiliated Hospital of Chengdu Medical College & Pidu District People’s Hospital, Chengdu, China

**Keywords:** intra-abdominal aggressive fibromatosis, pelvis, abscess, imaging, pathology

## Abstract

This study investigates the imaging diagnosis and differential diagnosis of intra-abdominal aggressive fibromatosis (IAF) complicated by acute infection. Aggressive fibromatosis is a rare mesenchymal tumor of unclear etiology, potentially associated with genetic factors, hormonal levels, trauma, and other factors. Due to its clinical rarity and nonspecific imaging manifestations, preoperative diagnosis is challenging and prone to misdiagnosis, particularly in the more complex presentation of IAF. We present a rare case of IAF accompanied by abscess formation, analyzing its imaging and pathological features alongside clinical manifestations, and discuss diagnostic and therapeutic strategies.

## Introduction

Aggressive Fibromatosis (AF), also known as Desmoid-type fibromatosis (DF), is a rare mesenchymal tumor originating from the connective tissue of muscle, fascia or aponeurosis. AF is defined by the World Health Organization (WHO) as a clonal fibroblast proliferative lesion that occurs in deep soft tissues, is characterized by invasive growth and has a tendency to local recurrence, but has no metastatic ability (1). The etiology of AF is unknown and may be related to genetics, hormone levels, trauma and surgery, among others. There are two types of AF, sporadic type and familial type. Most of them are sporadic and are caused by mutations in exon 3 of the gene encoding β-catenin. In addition, less than 15% of AF cases occur in patients with adenomatous colonic polyposis (APC) gene mutations, including familial adenomatous polyposis (FAP) and tumor susceptibility syndrome (2). According to the site of occurrence, it can be divided into abdominal wall type, extra-abdominal type and intra-abdominal type, among which intra-abdominal aggressive fibromatosis (IAF) is the least common (3). Symptoms of AF depend on the location and size of the tumor and can range from asymptomatic to severe pain, swelling, deformity, and loss of function (4). Patients with severe IAF can have complications such as intestinal obstruction, perforation, and bleeding (5). Here, we report a young female patient with IAF complicated with abscess. By analyzing the characteristics of imaging and pathology and combining relevant literature, we aim to improve the understanding of IAF and provide reference for clinical diagnosis and treatment decision-making.

## Case report

**Figure 1:**
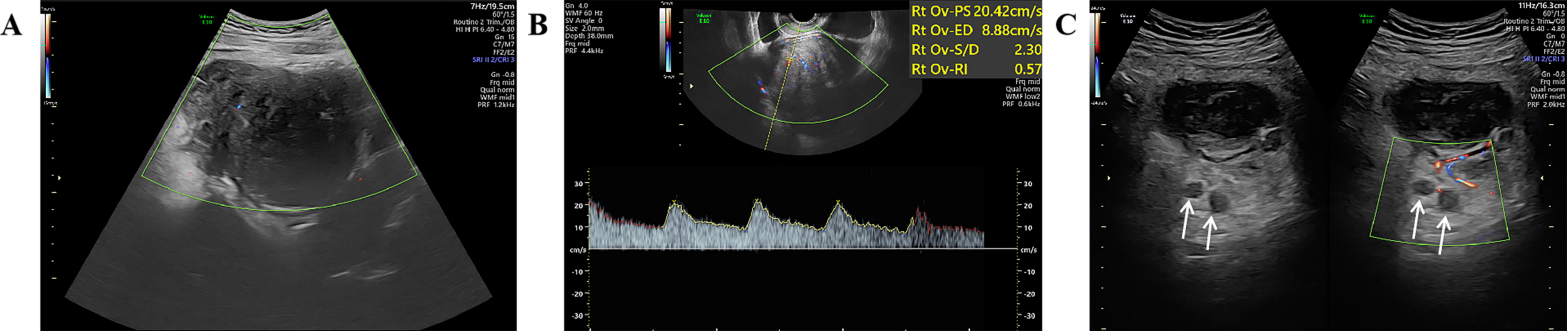
Ultrasound images; **(A)** Color Doppler showing punctate blood flow signals inside the hypoechoic mass; **(B)** Pulsed Doppler spectrum inside the hypoechoic mass; **(C)** Enlarged right abdominal lymph node (white arrow).

**Figure 2:**
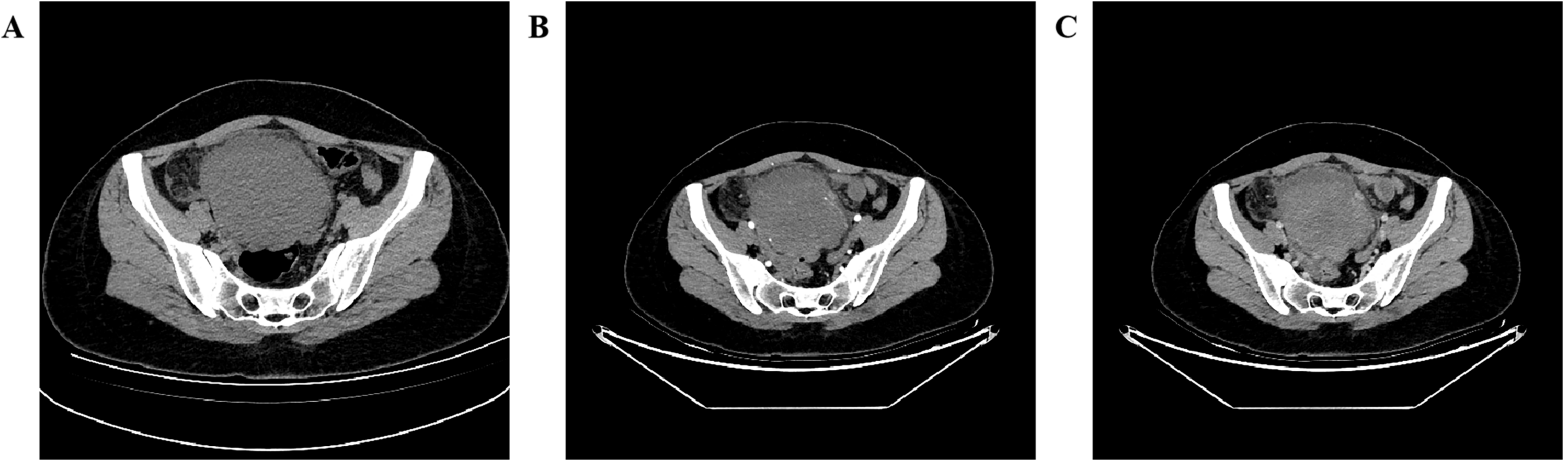
Pelvic CT axial view shows: (**A** plain scan, **B** enhanced arterial phase, **C** enhanced venous phase) A slightly hyperdense mass is seen in the pelvic cavity with ill-defined margins, blurred surrounding spaces, and heterogeneous enhancement, with more obvious enhancement in the upper part of the lesion.

**Figure 3:**
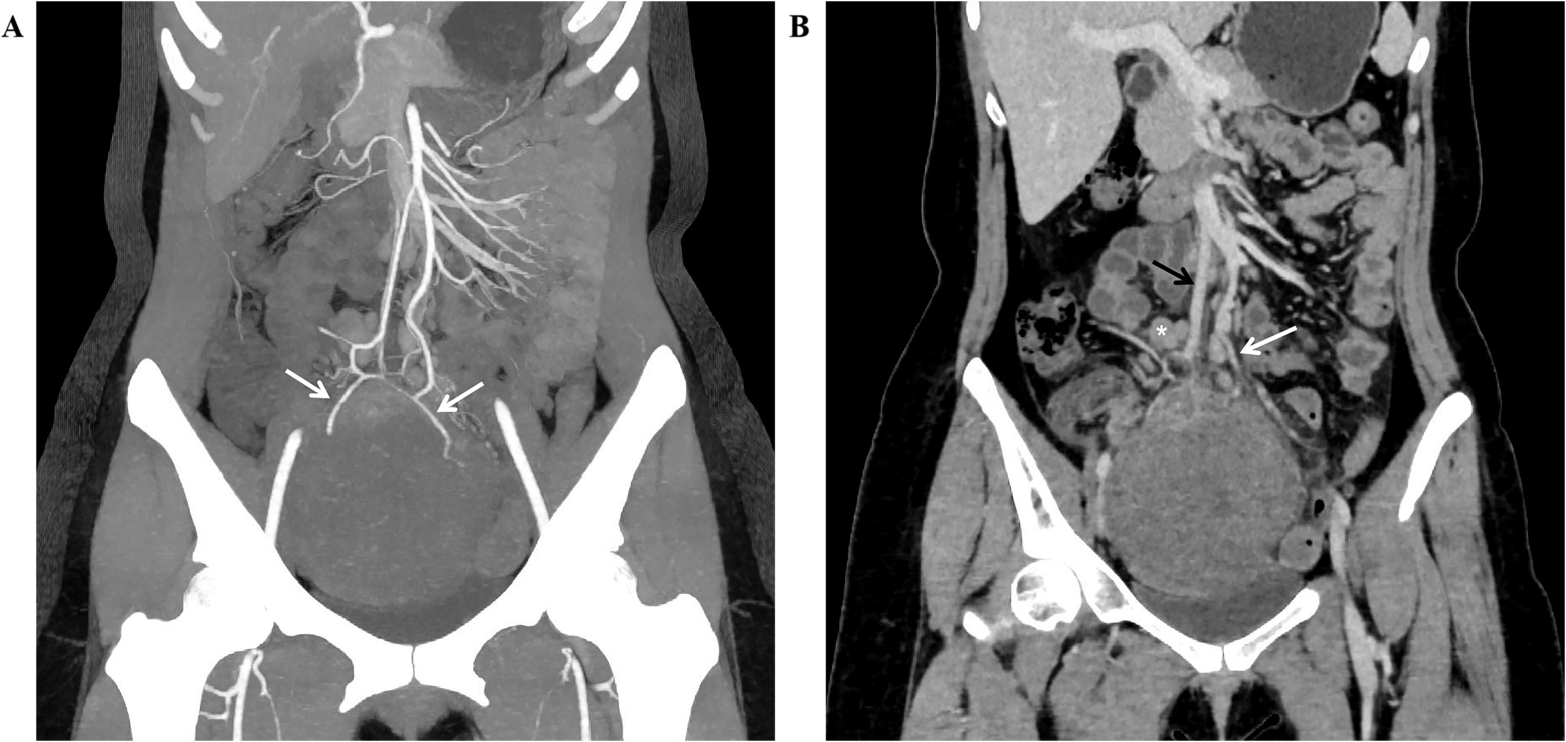
Coronal view of pelvic CT shows: (**A** enhanced arterial phase MIP image, **B** enhanced venous phase) the blood supply of the mass originates from the branches of the superior mesenteric artery (white arrow), and drains through the mesenteric vein (black arrow), with multiple enlarged lymph nodes (*) visible above the mass, A compressed small intestinal loop shadow can be observed between the mass and the bladder.

**Figure 4:**
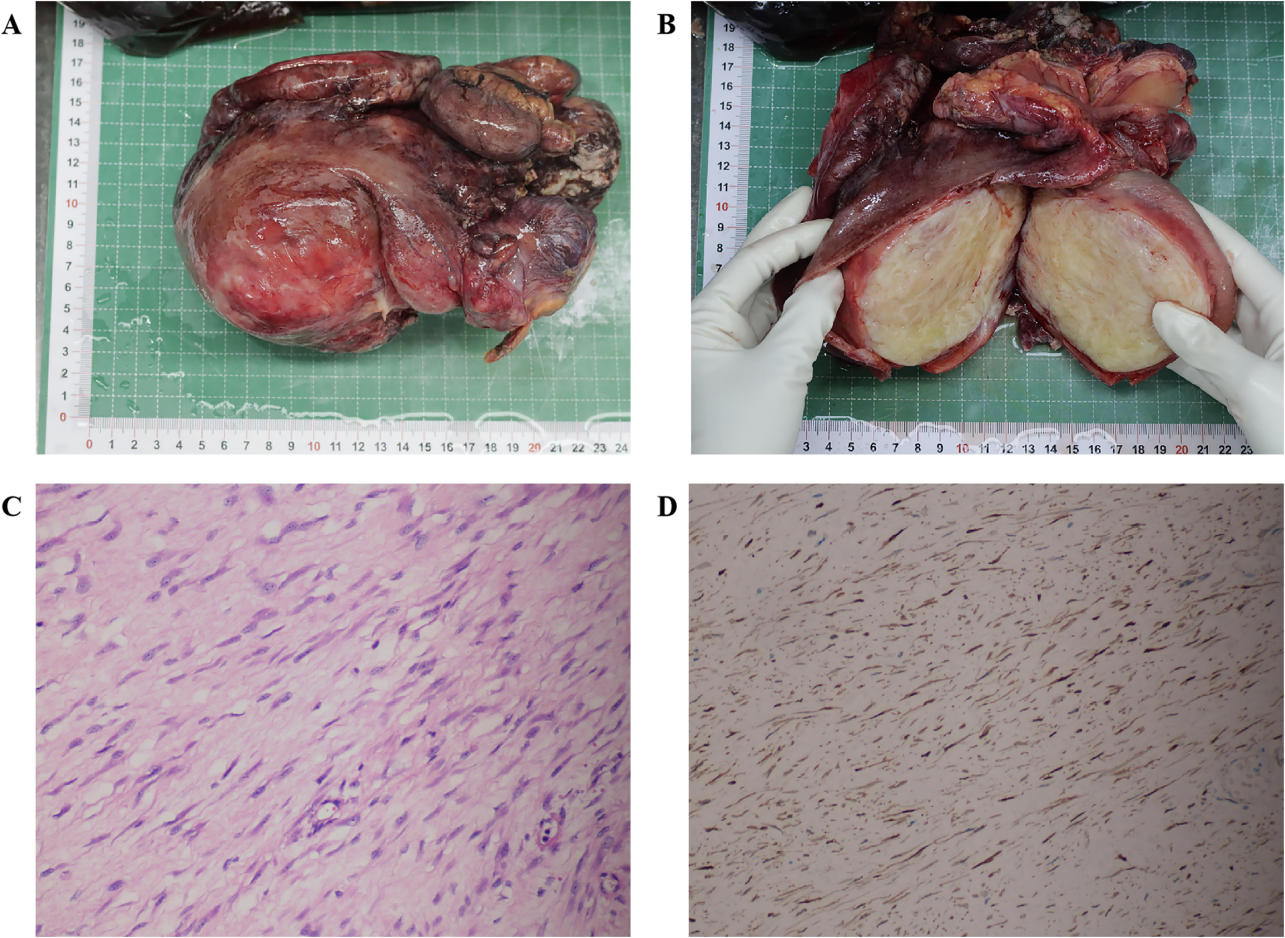
Gross tumor specimen examination, histopathological analysis, and immunohistochemical detection; **(A)** Image of the intact tumor and intestinal tract; **(B)** Image of the opened tumor; **(C)** Hematoxylin-eosin staining (magnification ×20); **(D)** Immunohistochemical analysis showing β-Catenin (nuclear +) (magnification ×20).

A 27-year-old woman came to the doctor because of intermittent pain in the lower abdomen for more than 1 month and fever 1 day ago. During this period, the symptoms were relieved after oral medication (details are unknown). A week ago, there was no obvious inducement for migratory abdominal pain, which transferred from the umbilicus to the right lower abdomen, and the pain worsened after changing the body position. Physical examination: obvious tenderness in the right lower abdomen, no rebound pain and muscle tension. A mass about 10 cm × 8 cm in size was palpable in the pelvic cavity, with unclear boundaries and inactive adhesions to the pelvic cavity. Blood routine examination: white blood cell count 13.1 (109/L)↑, neutrophil count 10.9 (109/L)↑, monocyte count 1.3 (109/L)↑, neutrophil ratio 82.7 (%)↑, lymphocyte ratio 8.7 (%)↓, acidophage ratio 0.0 (%)↓, high-sensitivity C-reactive protein 140.2 mg/L↑. Ultrasound examination: A hypoechoic mass with a size of about 11 cm × 8 cm ×10 cm was found in the pelvic cavity, The internal echo is uneven, with no obvious liquefied areas or calcification foci. The mass has a clear boundary and a relatively regular shape; CDFI: punctate blood flow signals were seen in it, PW: PS: 20.4 cm/s, S/D: 2.3, RI: 0.57. Two circular hypoechoic nodules were found in the right abdominal cavity, the larger one was about 13 mm × 13 mm (**Figure 1**). Ultrasound suggests: hypoechoic mass in pelvic cavity, considering neoplastic lesions, the source cannot be defined; Enlarged lymph nodes in the right abdominal cavity. CT examination revealed a slightly higher density mass in the pelvic cavity, about 10 cm × 9 cm in size, with a CT value of about 25HU. The edge was under smooth and the lesion enhancement was uneven. The local enhancement of the upper edge was obvious, and the overall enhancement was mild. The branch vessels of the superior mesenteric artery passed through it, the space around the lesion was blurred, and multiple enlarged lymph node shadows were seen. The mass is located above the bladder, anterior and superior to the uterus and closely adherent to it, with indistinct boundaries from the right adnexal region broussonetia papyrifera. The sigmoid colon is displaced cephalad and posteriorly (**Figures 2**, **3**). Between the mass and the bladder, compressed small bowel loops can be visualized. CT suggested: pelvic space-occupying lesions, adjacent multiple lymph nodes enlarged, and neoplastic lesions were considered. The patient underwent surgical treatment. During the operation, a huge mass was found in the ileocecal part, about 15 cm × 14 cm × 13 cm in size. The tumor was necrotic, and pus moss attached to the surface. The lesion involved part of the ascending colon and terminal ileum, and about 200ml of purulent exudate was seen in the abdominal cavity. Finally, the mass was completely removed, and part of the intestinal tube and omentum were removed. Pathological examination: naked eyes: a mass can be seen on the serosal surface of one side of the intestinal tube, which is closely connected with the intestinal tube, It has a size of 12 cm × 12 cm × 10 cm. The capsule of the mass is complete and smooth. The cut surface is gray and white. It is a solid mass with medium texture. The cut surface is fish - like and seems to have a mucus feeling, and bleeding and edema can be seen on the surrounding intestinal wall. Microscopically, the tumor boundary is unclear, infiltrating into the muscle layer of intestinal mucosa. The tumor cells are bundled and arranged in a woven way, and some areas are wavy. Collagen fibers can be seen to alternate, and some lymphocytes and neutrophils can be seen infiltrating. The morphology of the tumor cells is mild, slender spindle - shaped. The nucleus chromatin of the tumor cells is sparse or vacuolar, and some small nucleoli can be seen. Immunohistochemical results: β - Catenin (nuclear +), CD117 (–), CD34 (–), DOG - 1 (–), Desmin (partial +), Ki - 67 (+, ~ 5%), PHH3 (showing mitosis), S - 100 (–), SDHB (+), SMA (–), SOX - 10 (–) (**Figure 4**). Pathological diagnosis: Spindle cell tumor in the “giant tumor of ileocecal region”, combined with acute pyogenic inflammation of omentum. Combined with morphological and immunohistochemical results, it is consistent with IAF. After operation, the patient was transferred to ICU ward for symptomatic treatment such as anti - infection, nutritional support and fluid rehydration. On the 10th day after operation, pelvic CT re - examination showed that there were a small amount of exudative changes in the fat space in the operative area, thickening of mesangium, omentum and fascia, and a little localized gas accumulation. The patient was discharged on the 11th postoperative day. During the 3rd month of follow - up, pelvic CT showed that intra - abdominal pneumatosis and exudation in the operative area had been absorbed, and no recurrent imaging findings were found.

## Diagnosis and differential diagnosis

The imaging findings of AF lack specificity, and they vary due to its location and internal components. Preoperative diagnosis is difficult and preoperative diagnosis may be misdiagnosed. Extra abdominal and abdominal wall lesions are round - shaped masses or grow along the long axis of muscles (6, 7). IAF can appear as round - shaped soft tissue masses, and the edges of the lesions are usually unclear. Because it contains collagen and mucus components inside, CT usually shows a mass with uneven density, while enhanced scanning shows different degrees of enhancement, and most of the middle low - density areas have no obvious enhancement (8). CT examination can be used for postoperative follow - up of patients with intra - abdominal fibromatosis (9). MRI showed lesions with homogeneous isointensity on T1 - weighted images, similar to skeletal muscle, with non - homogeneous hyperintensity on T2 - weighted images and moderate enhancement on enhanced scans (3, 8).F18 - FDG PET/CT can indicate the response of patients treated with imatinib early (8). The ultrasound manifestations of AF are mostly hypoechoic solid masses with regular shape, clear boundaries and lacking blood supply. The changes of internal echoes depend on the proportion and distribution of components within the tumor. If IAF comes from the mesentery, because the tumor can pull and wrap the adjacent mesenteric tissue during the slow growth process, it shows the change of mesenteric tissue being phagocytosed by the tumor, that is, “mesenteric phagocytosis sign” (10). Hemorrhage, necrosis and cystic degeneration areas can occur inside larger tumors (11). In this case, contrast - enhanced CT showed that the branch vessels of the superior mesenteric artery passed through the pelvic mass, suggesting that the lesion may originate from the mesentery. However, ultrasound has certain limitations in analyzing the origin of the tumor due to its own imaging, and only suggests hypoechoic mass in the pelvic cavity.

AF is clinically rare. Although histologically benign, it has local invasiveness and a recurrence rate. The diagnosis of AF mainly depends on pathological diagnosis, which is gray - white to the naked eye and hard in texture, similar to scar tissue (9). Microscopically, tumor cells are composed of morphologically consistent fibroblasts and myofibroblasts. The tumor cells are slender spindle - shaped, and some areas can be stellate - shaped. The chromatin of tumor cell nuclei is sparse or vacuolar, which is characterized by myofibroblasts wrapped in collagen - rich matrix and vascular network lacking an envelope (8). Immunohistochemical staining analysis showed positive for β-catenin, vimentin, a-SMA, MSA, calponin and negative for CD34, CD117, h-caldesmon and S-100 proteins (9), among which β-catenin mutation analysis has been proposed as a specific tool for the diagnosis of AF (8).

IAF needs to be differentiated from other intra-abdominal tumors, such as gastrointestinal stromal tumors (GIST). CT images often show soft tissue masses with clear boundaries and uneven density, while low - density sites often suggest necrosis or bleeding. Magnetic resonance imaging usually shows uniform moderate enhancement on T1-weighted images, and if there is necrosis or hemorrhage, it presents as inhomogeneous high enhancement on T2-weighted images (12).The ultrasonic manifestations of GIST are related to the tumor volume. When the tumor is small, the sonogram mostly shows uniform hypoechoicity. As the tumor volume increases, the internal echo becomes heterogeneous, and hypo-anechoic areas can be seen (13). Cystic degeneration is one of the predictors of gastrointestinal stromal tumors with high malignant potential (14).GIST can usually express CD34, CD117, DOG-1, in immunohistochemical results, and some cases can also express S-100, SMA, while β-catenin is usually not expressed (15).It has been reported in the literature that GIST and AF may be susceptible and can be combined (16). The imaging features of solitary fibrous tumor (SFT) in the abdominal and pelvic cavity are mainly large, well - bounded masses with rich blood vessels and varying degrees of necrosis, cystic degeneration or hemorrhage. On T2WI, they usually show uneven signals. The high - signal area and low - signal area represent flow empty, fibrosis or collagen respectively (17).The immunohistochemical results of SFT generally express STAT6, CD34, and Bcl-2 (18). Primary gastrointestinal lymphoma has similar imaging findings to IAF. Although some imaging features such as lymphadenopathy and thickening of adjacent intestinal wall suggest lymphoma more, they are not specific and must be analyzed histopathologically. In histology, the most significant finding of gastrointestinal lymphoma is the existence of different numbers of lymphoepithelial lesions and obvious infiltration of tumor cells into mucosal glands. Mature B cell tumors can express CD19, CD20, CD79a, but not CD5, CD10, CD23, BCL-6 (19).

## Discussion

IAF accounts for about 10% of all AF(5), while IAF with abscess formation is even rarer. In this case, the huge IAF is accompanied by an abscess, which may be caused by the complex anatomical structure of ileocecal part (the existence of ileocecal valve and appendix), the long residence time of intestinal contents, the rapid reproduction of intestinal bacteria, and the potential condition of inflammation (20). At the same time, due to the large space in abdominal cavity, the tumor expands and grows, which compresses the surrounding intestinal tube, causes intestinal peristalsis to slow down and intestinal contents to be accumulated, and then causes intestinal ischemia and mucosal barrier damage (21). In this case, abscess-related infection symptoms (fever, significantly elevated white blood cells and CRP, and empyema abdominal cavity) are one of the outstanding manifestations, which increases the complexity of preoperative diagnosis of IAF and easily misleads the diagnostic direction. Clinicians should be vigilant. This is related to the close connection between imaging manifestations and pathological features: on imaging, tumors in an inflammatory background may present with blurred boundaries of the lesion area, accompanied by signs of exudation or edema (such as blurred fat spaces around the lesion visible on CT). During enhanced scanning, they may also show heterogeneous enhancement due to the combined effect of inflammatory hyperemia and tumor neovascularization, further interfering with diagnostic judgment.

It is worth noting that the diagnosis process of this patient reflects the importance of multidisciplinary collaboration (22). Clinical manifestations and laboratory examinations suggest infectious diseases, and imaging examinations (ultrasound, CT) clarify the existence of huge pelvic space occupation and its relationship with surrounding structures, but it is difficult to accurately judge the nature and histological origin of the tumor. The CT sign of “superior mesenteric artery branch vessels passing through it” is helpful to suggest IAF, because IAF can originate in the mesentery and grow around blood vessels. Additionally, we observed compressed and displaced small intestinal shadows in the space between the mass and the bladder, which further indicated the origin of the mass and held significant implications for the diagnosis of IAF. Although magnetic resonance imaging (MRI) and PET-CT also have potential roles in diagnosing IAF, unfortunately, due to the prolonged waiting time for these examinations, only ultrasound and CT scans were completed in this case before emergency surgical treatment was performed. Of course, the gold standard of diagnosis still depends on pathological examination, especially the immunohistochemical results of positive β-Catenin nucleus, which is the key basis for the diagnosis of IAF. This suggests that for young patients, when finding a huge solid space closely related to mesenteric vessels in the abdominal cavity, and even if it is complicated with obvious infection, the possibility of IAF should be considered.

Due to the heterogeneity of biological behavior of AF, the choice of treatment plan needs to be considered comprehensively. It is pointed out in the literature that surgery is the main means of treatment for AF patients, and postoperative radiotherapy can reduce the local recurrence rate. Close observation, drug treatment or radiotherapy can also be considered (15). Drug therapy is particularly suitable for cases where the lesion involves adjacent vital organs or cannot be completely resected surgically (23). The drug treatment regimen for AF mainly includes nonsteroidal anti-inflammatory drugs, anti-estrogen drugs, chemotherapeutic agents, and targeted drugs such as imatinib (15). Given the special location of the tumor, IAF is not suitable for radiotherapy (23). Patients with AF located in the mesentery or head and neck can develop life - threatening complications requiring more aggressive treatment (15). This patient underwent ileocecal resection and partial omentum resection, and the tumor margin was negative. He was treated with antibiotics due to postoperative infection. He recovered well after operation, but did not undergo radiotherapy and chemotherapy. This patient has no signs of recurrence in the short term after operation, so follow - up observation can be continued.

## Conclusion

This case reports a rare young female patient with ileocecal IAF complicated with abscess. Its main clinical manifestations are abdominal pain and fever. Laboratory examination suggests severe infection, and imaging examination shows that there is a huge space - occupying lesion in the pelvic cavity. The diagnosis was confirmed by surgical resection and postoperative pathological examination. After complete surgical resection of the tumor and the affected intestinal canal, supplemented by active anti - infection and supportive treatment, the patient recovered well, and no recurrence was found during short - term follow - up. This case suggests that the possibility of IAF should be considered for young patients with huge intra - abdominal space - occupying lesions, even if they are combined with obvious infection. Imaging examination is of great value in locating and evaluating the relationship between tumors and surrounding structures, and pathological examination along with β - Catenin immunohistochemistry results is the gold standard for diagnosis. Surgical resection combined with perioperative comprehensive management is an effective means to treat such complex IAF, and long - term follow - up monitoring for recurrence is essential. This case enriches the clinical manifestations of IAF and enhances our understanding of IAF complicated by infectious complications. By sharing this case, we hope to provide valuable reference for clinicians and further promote the research, diagnosis and treatment level of invasive fibromatosis, a rare disease.

## Data Availability

The datasets presented in this study can be found in online repositories. The names of the repository/repositories and accession number(s) can be found in the article/supplementary material.
